# External Evaluation of Population Pharmacokinetic Models to Inform Precision Dosing of Meropenem in Critically Ill Patients

**DOI:** 10.3389/fphar.2022.838205

**Published:** 2022-05-18

**Authors:** Nan Yang, Jing Wang, Yueliang Xie, Junjie Ding, Cuifang Wu, Jingjing Liu, Qi Pei

**Affiliations:** ^1^ Department of Pharmacy, The Third Xiangya Hospital, Central South University, Changsha, China; ^2^ Department of Pharmacy, Xiamen Children’s Hospital (Children’s Hospital of Fudan University Xiamen Branch), Xiamen, China; ^3^ Center for Tropical Medicine and Global Health, Nuffield Department of Clinical Medicine, University of Oxford, Oxford, United Kingdom; ^4^ Department of Intensive Medicine, The Third Xiangya Hospital, Central South University, Changsha, China

**Keywords:** meropenem, population pharmacokinetics, external evaluation, critically ill patients, Bayesian forecasting

## Abstract

Routine clinical meropenem therapeutic drug monitoring data can be applied to model-informed precision dosing. The current study aimed to evaluate the adequacy and predictive capabilities of the published models with routine meropenem data and identify the dosing adaptations using a priori and Bayesian estimation. For this, 14 meropenem models for the external evaluation carried out on an independent cohort of 134 patients with 205 meropenem concentrations were encoded in NONMEM 7.3. The performance was determined using: 1) prediction-based and simulation-based diagnostics; and 2) predicted meropenem concentrations by a priori prediction using patient covariates only; and Bayesian forecasting using previous observations. The clinical implications were assessed according to the required dose adaptations using the meropenem concentrations. All assessments were stratified based on the patients with or without continuous renal replacement therapy. Although none of the models passed all tests, the model by Muro et al. showed the least bias. Bayesian forecasting could improve the predictability over an a priori approach, with a relative bias of −11.63–68.89% and −302.96%–130.37%, and a relative root mean squared error of 34.99–110.11% and 14.78–241.81%, respectively. A dosing change was required in 40.00–68.97% of the meropenem observation results after Bayesian forecasting. In summary, the published models couldn’t adequately describe the meropenem pharmacokinetics of our center. Although the selection of an initial meropenem dose with a priori prediction is challenging, the further model-based analysis combining therapeutic drug monitoring could be utilized in the clinical practice of meropenem therapy.

## Introduction

Meropenem, a broad-spectrum carbapenem antibiotic, has currently gained a predominant position in the treatment of severe infections in critically ill patients due to its strong antibacterial activity, low toxicity, and fast distribution in the body (Leroy et al., 1992; Nicolau, 2008; Breilh et al., 2013). The physiological and pathological conditions in critically ill patients cause significant β-lactam pharmacokinetic (PK) and pharmacodynamics (PD) variability (Gonçalves-Pereira and Póvoa, 2011; Roberts et al., 2014a). Notably, wide fluctuation in renal function, ranging from augmented renal clearance to renal failure or eventually to continuous renal replacement therapy (CRRT), may further alter the PK of meropenem. Especially for CRRT patients, the volume of distribution reported based on the published meropenem models was found to be higher compared with the healthy volunteer (Chaijamorn et al., 2020), and meropenem could also be eliminated by CRRT owing to its hydrophilicity, small molecule, and low protein binding (Li et al., 2020).

Bactericidal activity of meropenem depends on the percentage of the dosing interval over which its free plasma concentration remains above the minimum inhibitory concentration (ƒT > MIC) (Drusano, 2004), and recent studies recommend a more appropriate target of 100% ƒT>4×MIC for the critically ill (Roberts et al., 2010b; Udy et al., 2012). At present, approximately 40% of severely ill patients fail to achieve the target of trough concentrations above the MIC for 100% of the dosing interval, and several prospective studies have also shown that meropenem measurements fail to achieve the cut-off values (Roberts et al., 2014b). In view of the variable and unpredictable meropenem pharmacokinetics, therapeutic drug monitoring (TDM) has emerged as a potential approach to optimize high targets and improve clinical outcomes. Although monitoring the β-lactam concentrations is recommended by several guidelines in the intensive care unit (ICU) (Roberts et al., 2010a; Dellinger et al., 2013), the collection of meropenem samples could be limited due to the busy clinical environment and ethical considerations, which may not allow essential guidance of an initial dose and a good appreciation of pharmacokinetic characteristics. Currently, an attractive approach of model-informed precision dosing, mainly referring to population pharmacokinetic (popPK) models, is proposed to adjust the dose individually (Keizer et al., 2018). The popPK method quantitatively analyzes intensive blood specimens and sparse TDM data from diversified populations and explains between- or within-subject variability. Moreover, a popPK analysis can be used to inform the first dose before drug administration with an a priori approach, that is, probabilistic dosing, and can be applied to the dosage adaptations with Bayesian forecasting. In other words, model-based Bayesian analysis combined with TDM programs would be more forgiving and compatible in daily practice.

To date, a few external evaluation studies of meropenem have been conducted to assess the published model’s predictive performance. However, the above studies were performed in different classes of renal function without the inclusion of CRRT patients, and the adequacy of the meropenem models was not assessed before (Wong et al., 2015; Tamatsukuri et al., 2018; Dhaese et al., 2019; Wang et al., 2020). Furthermore, given the challenges of conventional trough concentration-guided dosing, the question arises as to how to utilize the model-based analysis to inform the selection of an initial dose and adaptation of subsequent doses in the individualization of meropenem therapy during the TDM process.

Thus, the current study aimed to identify: 1) the predictability and adequacy of meropenem popPK models in an external cohort, including patients on CRRT; and 2) the dose adaptation utilized in meropenem therapy with an a priori prediction approach and Bayesian forecasting.

## Materials and Methods

### Reviews of Published PopPK Analyses

The databases (PubMed, Web of Science, and Embase) were systematically searched for population pharmacokinetic analysis of meropenem published up to July 2021. Key terms such as “[meropenem] AND (pharmacokinetic* OR population model) AND (critical care OR intensive care)” were applied as a search strategy, and the reference lists of the identified publications were also screened for additional relevant articles. The models were included if: 1) the study was a population analysis of meropenem in critically ill patients and 2) the publications were in English. The models were excluded if: 1) the studied population was patients receiving extracorporeal membrane oxygenation; 2) the modeling datasets were overlapped or the articles were duplicated; 3) model parameters were not available for external evaluation; and 4) the publications were reviews or methodological articles.

### External Evaluation Study Cohort

A dataset of 134 ICU patients with 205 meropenem samples (45 CRRT patients with 85 concentrations and 89 no-CRRT patients with 120 concentrations) was obtained from the Third Xiangya Hospital of Central South University, Changsha, China, from April 2016 to August 2017. Patients received 0.5–2 g of meropenem q4∼12 h over a 0.5–4 h infusion and experienced clinical routine TDM of meropenem was included. Of these, 88 patients had a single PK sample, and 46 patients had at least two observations. After the exclusion of seven values of lower limits of quantitation (0.5 mg/L) points, the measured meropenem levels ranged from 0.53 to 106.4 mg/L, and the 25th and 75th quartiles of the measured meropenem concentration were 5.10 and 31.80 mg/L, respectively. In addition, 30.3 and 57.55% of meropenem samples did not reach the target concentrations (8–45 mg/L) in the CRRT and non-CRRT patients, where the chosen target was 100% ƒT>4×MIC with a cut-off value of 2 mg/L for *Pseudomonas aeruginosa* (European Committee of Antimicrobial Susceptibility Testing, http://www.eucast.org/), and the trough levels above 45 mg/L were defined as the toxic levels (Imani et al., 2017). The creatinine clearance calculated using the Cockcroft–Gault equation was in the wide range of 3.4–217.5 ml/min. Further detailed demographic and clinical characteristics recorded from an electronic medical record system are listed in Table 1. The study protocols were approved by the Ethics Committee of the Third Xiangya Hospital of Central South University. Importantly, no patient data in the external evaluation cohort was previously used in the development of any other models.

**TABLE 1:** Clinical characteristics of the external dataset.

Characteristics	Values^a^
Number of patients (male/female)	134 (83/51)
Patients undergoing CRRT	45 (33.58%)
Patients not undergoing CRRT	89 (66.42%)
Age (years)	58 (22–89)
Height (cm)	166 (148–175)
Body weight (kg)	58.50 (40–84)
BMI (kg/m^2^)	22.60 (15.57–29.07)
Serum albumin (g/L)	29.10 (16.70–54)
Serum creatinine (μmol/L)	143 (24–1,145)
Creatinine clearance (ml/min)^b^	40 (3.40–271.50)
24 h fluid input (ml)	3,754.50 ± 1,477.80
24 h fluid output (ml)	3,297.90 ± 1898.20
24 h urine output (ml)	2077.70 ± 1,633.70
Dosage	
1 g Q8h	57.30%
1 g Q6h	12.90%
0.5 g Q8h	8.60%
2 g Q8h	6.50%
2 g Q12h	4.30%
Others	10.40%
Number of samples	
In patients undergoing CRRT	85(41.46%)
In patients not undergoing CRRT	120(58.54%)

a Shown as mean ± SD, number, or %.

b Calculated from serum creatinine using the Cockcroft–Gault formula.

On attaining a steady state in each patient, a 2 ml arterial blood sample was withdrawn. Blood samples were sent to the core laboratory of the Department of Pharmacy and stored at 4°C for no longer than 24 h. After the sample preparation for protein precipitation (acetonitrile), the meropenem concentrations were quantified by the high-performance liquid chromatography-tandem mass spectrometer method using Agilent Eclipse XDB-C18 (2.1 mm × 150 mm, 5 µm) with the mobile phase A of water with 0.2% formic acid and mobile phase B of methanol with 0.1% formic acid. The lower limit of quantification was 0.5 mg/L, and the coefficients of variation of inter-day and intra-day precisions were within 15%, with a calibration range of 0.5–100 mg/L.

### Overall Model Evaluation (Population Level)

**FIGURE 1:**
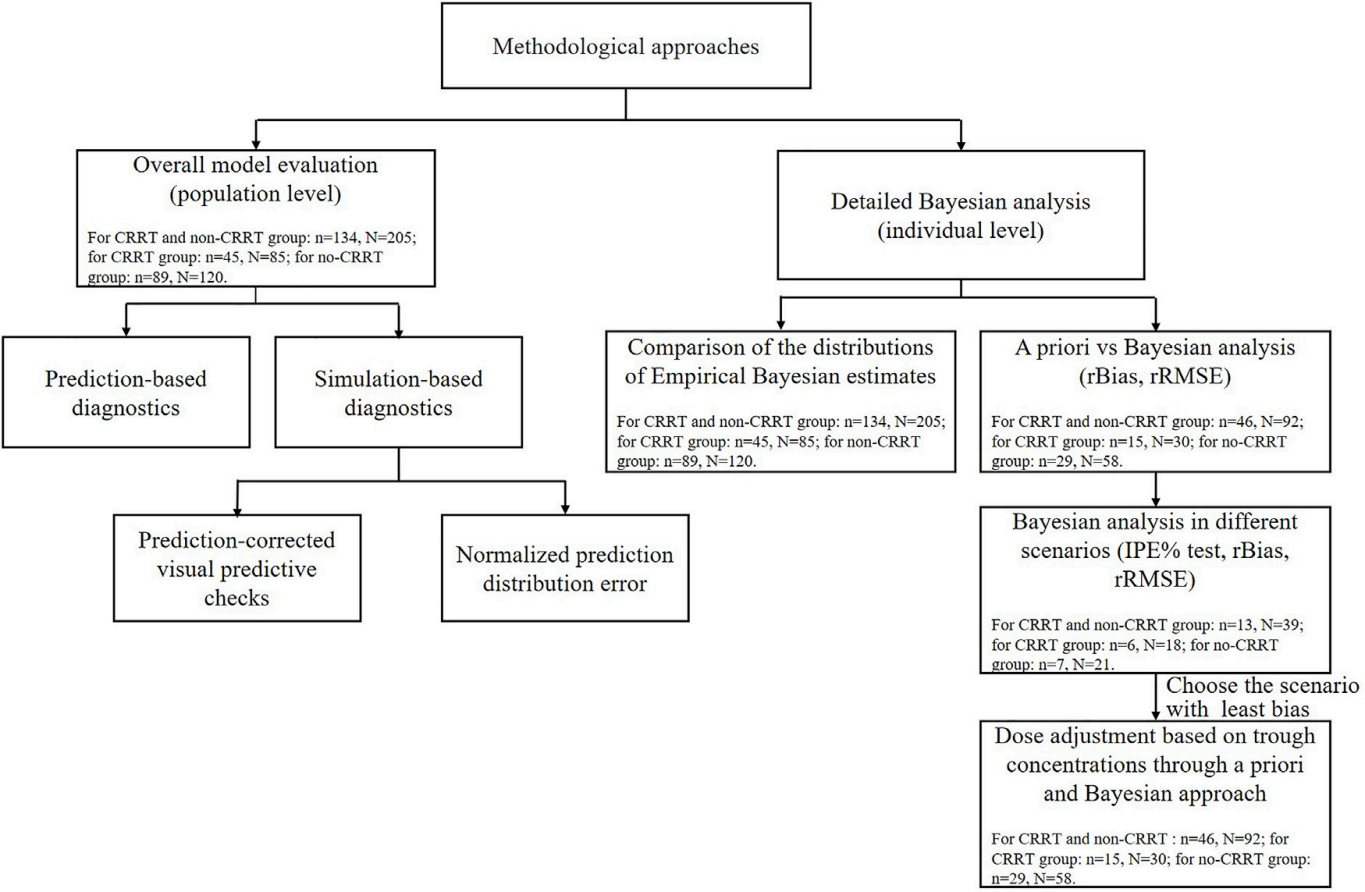
The methodological steps and data used for the external evaluation study. N and n represent the meropenem samples and critically ill patients used in each step. IPE, rBias, and rRMSE represent the individual prediction error, relative bias, and relative root mean squared error, respectively.

The external evaluation was performed using the nonlinear mixed-effects modeling tool NONMEM 7.3 (ICON Development Solutions, United States) compiled with GFortran 4.6, and the output was displayed using R studio (version 3.6.1, http://www.r-project.org). The popPK models were re-established by compiling formulas and parameters into the control file and executing with an iteration of 0 (MAXEVAL = 0). All assessments of predictive performance were stratified based on the studied patients with or without CRRT therapy. Detailed methodological steps of evaluation and the data used in each step are shown in Figure 1.

#### Prediction-Based Diagnostics

The relative prediction error (PE%, Eq. 1) of each model was estimated by comparing the population predicted concentrations (PRED) and the corresponding observations (OBS) for each subject in the external dataset. The median prediction error (MDPE, median PE%) and median absolute prediction error (MAPE, median |PE|%) were used to evaluate the total deviation and precision, respectively. The composite indicators of F_20_ and F_30_, defined as the percentages of prediction errors falling within 20 and 30%, were used to simultaneously characterize accuracy and precision. Model appropriateness was confirmed when the following results were obtained: MDPE ≤ ±20%, MAPE ≤30%, F_20_ ≥ 35%, and F_30_ ≥ 50% (Sheiner and Beal, 1981; Li et al., 2021). Moreover, population-predicted concentrations were plotted against the meropenem observations to assess the model’s global fit.

$$\text{P}\text{E}\%=\frac{C_{PRED}-C_{OBS}}{C_{OBS}}\times 100\%$$ (1)

### Simulation-Based Diagnostics

Prediction-corrected visual prediction checks (pcVPC) and normalized prediction distribution error (NPDE) were performed based on 1,000 Monte Carlo simulated datasets generated using the final identified models and displayed using the “vpc” R package (version 1.2.2, https://cran.r-project.org/package = vpc.) and the NPDE add-on R package (version 2.0, https://cran.r-project.org/package=npde), respectively. The model global fit of the identified models was visually assessed by overlaying the 90% confidence interval of 5, 50, and 95% quantiles for the simulations with the corresponding quantiles for the meropenem concentrations in different subpopulations. Details of the NPDE test are presented in Supplementary Appendix S1.

### Detailed Bayesian Forecasting Analysis (Individual Level)

#### Comparisons of Empirical Bayesian Estimation

Model adequacy was evaluated by comparing discrepancies between the empirical Bayesian estimate (EBE) of the theoretical distribution and the estimated distribution. The estimated distribution of the pharmacokinetic parameters for each patient was estimated with the full dataset of meropenem after performing the post hoc analysis, whereas the theoretical distributions of the parameters were captured based on the reported parameters without meropenem observations. The η values from the “phi” files, which refer to the EBEs of the parameter, were identified and extracted. Then, the probability density of the EBEs for each model was plotted in the R studio.

#### Bayesian Analysis

To evaluate the performance of a priori prediction, the meropenem concentrations in the dataset were predicted using solely patient covariates and were estimated by the Bayesian approach with previous meropenem concentrations. The differences between the two approaches were compared by calculating the relative bias (rBias, bias (2)) and relative root mean squared error (rRMSE, precision (3)) at the individual level:

$$\text{r}\text{B}\text{i}\text{a}\text{s}=\frac{1}{\text{N}}\overset{i}{\underset{1}{\sum }}\frac{{\text{C}}_{\text{p}\text{r}\text{e}\text{d},\;\text{i}}-{\text{C}}_{\text{o}\text{b}\text{s}\text{e}\text{r}\text{v}\text{e}\text{d},\;\text{i}}}{\left({\text{C}}_{\text{p}\text{r}\text{e}\text{d},\;\text{i}}+{\text{C}}_{\text{o}\text{b}\text{s}\text{e}\text{r}\text{v}\text{e}\text{d},\;\text{i}}\right)/2}\times 100$$ (2)

$$\text{r}\text{R}\text{M}\text{S}\text{E}=\sqrt{\frac{1}{\text{N}}\overset{i}{\underset{1}{\sum }}\frac{{\left({\text{C}}_{\text{p}\text{r}\text{e}\text{d},\;\text{i}}-{\text{C}}_{\text{o}\text{b}\text{s}\text{e}\text{r}\text{v}\text{e}\text{d},\;\text{i}}\right)}^{2}}{{\left(\left({\text{C}}_{\text{p}\text{r}\text{e}\text{d},\;\text{i}}+{\text{C}}_{\text{o}\text{b}\text{s}\text{e}\text{r}\text{v}\text{e}\text{d},\;\text{i}}\right)/2\right)}^{2}}}\times 100$$ (3)

where N is the number of meropenem observations, and i represents the *i*th value, respectively. The models with lower rRMSEs were considered unbiased if their rBias were within the range of ±20% (Sheiner et al., 1979).

The detailed influence of prior observations in the Bayesian method was investigated with a subset of patients with ≥3 observations (n = 13). For each patient, the individual prediction (IPRED) of the third dosing interval was predicted and compared with the corresponding observation using different combinations (Eq. 4): 1) a priori, 2) the most recent (second) only, and 3) the first and second. MDIPE (median IPE%), MAIPE (median absolute IPE%), IF_20_ (IPE% within ±20%), and IF_30_ (IPE% within ±30%) and rBias and rRMSE were double checked for the predictive performance.

$$\text{I}\text{P}\text{E}\%=\frac{C_{IPRED}-C_{OBS}}{C_{OBS}}\times 100\%$$ (4)

As meropenem efficacy and clinical decisions were ultimately related to the concentrations, the clinical applicability was assessed by the need for dose adjustment when used in the dosing event with the least bias using Bayesian and a priori methods. The chosen meropenem target concentration of 8–45 mg/L for *P. aeruginosa* was listed before. The next dose to be adjusted was classified into three categories according to the predicted and observed values: 1) meropenem concentrations <8 mg/L, increase; 2) 8 ≤ meropenem concentrations ≤45 mg/L, maintain; and 3) meropenem concentrations >45 mg/L, decrease.

## Results

### Reviews of Published PopPK Analyses

After systematic research, a total of 14 popPK studies of meropenem in severely ill patients were finally identified and extracted from the original article (Li et al., 2006; Roberts et al., 2009; Crandon et al., 2011; Muro et al., 2011; Jaruratanasirikul et al., 2015; Ulldemolins et al., 2015; Mattioli et al., 2016; Burger et al., 2018; Sjövall et al., 2018; Dhaese et al., 2019; Ehmann et al., 2019; Padullés Zamora et al., 2019; Grensemann et al., 2020; Onichimowski et al., 2020). Among them, four models used data from patients with and without CRRT therapy (Li et al., 2006; Muro et al., 2011; Jaruratanasirikul et al., 2015; Burger et al., 2018), six from patients without CRRT therapy (Roberts et al., 2009; Crandon et al., 2011; Mattioli et al., 2016; Sjövall et al., 2018; Dhaese et al., 2019; Ehmann et al., 2019), and four from patients with CRRT therapy (Ulldemolins et al., 2015; Grensemann et al., 2020; Padullés Zamora et al., 2019; Onichimowski et al., 2020). More than half of the studies were conducted in Europe, and only two studies were conducted in Asia (Muro et al., 2011; Jaruratanasirikul et al., 2015). The sample sizes of the enrolled models varied from 9 to 101, and the HPLC method was applied in all studies to determine the plasma concentration of meropenem (Supplementary Table S1).

The included meropenem models differed with respect to the structural models (one-compartment models: n = 5 and two-compartment models: n = 9), significant covariates, and population heterogeneity (Table 2). Creatinine clearance or other markers of renal function such as residual diuresis were the most recurrent significant covariates (10/14), determining consensus on the consideration of renal function when treated with meropenem. More than three models identified body weight or serum albumin concentration as key predictors of the apparent volume of distribution (Vd) (Li et al., 2006; Crandon et al., 2011; Ulldemolins et al., 2015; Mattioli et al., 2016; Burger et al., 2018; Ehmann et al., 2019; Onichimowski et al., 2020). Additionally, model-specific covariates were considered to perfectly characterize meropenem pharmacokinetics.

**TABLE 2:** Summary of published population pharmacokinetic studies of meropenem in critically ill patients.

Study	Structure Model	Pharmacokinetic Parameters and Formulas				Covariates Retained in the Final Model
		Formula	CL (L/h)	V/V1 (L)	Model Variability (%)	
Roberts et al.^c^	2CMT	TVCL(L/h) = θ_1_×CLcr^a^	13.6	7.9	IIV_(CL)_ = 15.3 IIV_(V)_ = 44.7	CLcr^a^
Ulldemolins et al.^d^	1CMT	CL(L/h) = θ_CL_+0.22× (residual diuresis/100)	3.68	33.00	IIV_(CL)_ = 37.0 IIV_(V)_ = 45.0	Residual diuresis, BW
		V(L) = θ_V_ × (WT/73)^2.07^				
Burger et al	2CMT	CL_CRRT_ (L/h) = CL_res_ + S_c_ × Q_FD_	CL_CRRT_: 4.8 CL_no-CRRT_: 8.0	17.00	IIV_(CL)_ = 40.0 IIV_(V)_ = 51.0	CLcr^a^, BW
		CL_res_(L/h) = 3.2				
		CL_no-CRRT_(L/h) = 5.90 × [1 + 0.0071(CL_cr_ ^a^- medianCL_cr_ ^a^/median CL_cr_ ^a^)]				
		V_C_(L) = 16 × (BW/medianBW) × 1.7				
		Q(L/h) = 14				
		V_P_(L) = 15				
Ehmann et al.^c^	2CMT	When CLCR_CG_ < CLCR_CG_-INF	9.25	7.89	IIV_(CL)_ = 27.1 IIV_(V)_ = 31.5	CLcr^a^, BW, ALB
		CL-CLCR_CG_ (L/h) = θ_CL_ × [1 + 0.00977 × (CLCR_CG_-80.8)]				
		When CLCR_CG_ > CLCR_CG_-INF				
		CL- CLCR_CG_ (L/h) = CL-INF.				
		V1-WT(L) = θ_V1_ ×(WT/70)^0.945^				
		V2-ALB(L) = θ_V2_×[1–0.202×(ALB-2.79)]				
		Q(L/h) = 28.4				
Jaruratanasirikul et al	1CMT	CL = TVCL × e^η1^	3.01	23.7	IIV_(CL)_ = 48.0 IIV_(V)_ = 35.0	CLcr^b^
		TVCL=(θ_1_+θ_2_ × MDRD CLcr)				
		V = TVV × e^η2^				
Padullés et al.^d^	2CMT	CL(L/h) = 0.702 × FR	7.78	24.9	IIV_(CL)_ = 50.79 IIV_(V)_ = 45.70	NA
		Vc(L) = 24.9				
		V_p_(L) = 283				
		CL_D_(L/h) = 6.49				
Muro et al	1CMT	CL(L/h) = 11.1×(mSCR/0.7)^−1^	11.1	33.6	IIV_(CL)_ = 52.1	mSCR
Crandon et al.^a^	2CMT	K10 = 0.3922 + 0.0025× CLcr	NA	0.239	IIV_(V)_ = 53.76	CLcr, Adjbw
		V_1_ = AdjBW (kg) × 0.239 L				
Li et al	2CMT	CL (L/h) = 14.6 ×(CLcr/83)^0.62^ × (AGE/35)^−0.34^	14.6	10.8	IIV_(CL)_ = 34.3 IIV_(V)_ = 31.94	CLcr Age, WT
		V_C_(L) = 10.8 × (WT/70)^0.99^				
		Q (L/h) = 18.6				
		V_P_ (L) = 12.6				
Dhaese et al.^c^	1CMT	CL = TVCL× (CG-CLCR/135)	9.46	48.1	IIV_(CL)_ = 37.5	CLcr
Mattioli et al.^c^	1CMT	CL = θ_1_× (1 ± θ_4_) × (1 ± θ_6_) ×η_1_	2.181	8.305	IIV_(CL)_ = 44.38 IIV_(V)_ = 66.48	Sepsis, ALB Age, sex
		V = θ_2_× (ALB/22) ^θ3^× (AGE/61) ^θ5^×η_2_				
Onichimowski et al.^d^	2CMT	V_1_ = 27.9(ALB/24.6) ^−2.87^× exp(η_V1_)	15.1	27.9	IIV_(CL)_ = 43.7 IIV_(V)_ = 53.1	ALB
Grensemann et al.^d^	2CMT	NA	5.06	8.31	IIV_(CL)_ = 29.8	NA
Sjövall et al.^c^	2CMT	CL = TVCL×[2+(CLcr×0.083)]	6.83	16.916	IIV_(CL)_ = 40.578 IIV_(V)_ = 38.872	CLcr

CMT, compartment; TVCL/θ_CL_, typical value of clearance; IIV, interindividual variability; θ_V_/TVV, typical value of V; BW/WT, body weight; Vc/V1, central volume of distribution; CL_CRRT_, total meropenem clearance in patients undergoing CRRT; CL_res_, meropenem residual clearance in CRRT, patients; S_c_, sieving coefficient; Q_FD_, FD, flow; FD, filtrate–dialysate; CLCR_CG_, the Cockcroft–Gault creatinine clearance; CL-CLCR_CG_, CLCR_CG_, effect on CL; CLCR_CG_-INF, CLCR_CG_, value serving as an inflection point; V1-WT, WT, effect on V1; ALB, serum albumin concentration; MDRD, modification of diet in renal disease; FR, flow rate calculated as the sum of dialysate and ultrafiltrate flow rates; CL_D_, distributional CL, between central and peripheral compartments; NA, not available; mSCR, modified serum creatintine; AdjBW, adjusted body weight.

a Creatintine clearance is calculated with the Cockcroft–Gault formula.

b Creatintine clearance is calculated with the Modification of Diet in Renal Disease formula.

c Non-CRRT models.

d CRRT models.

### Overall Model Evaluation (Population Level)

#### Prediction-Based Diagnostics

**FIGURE 2:**
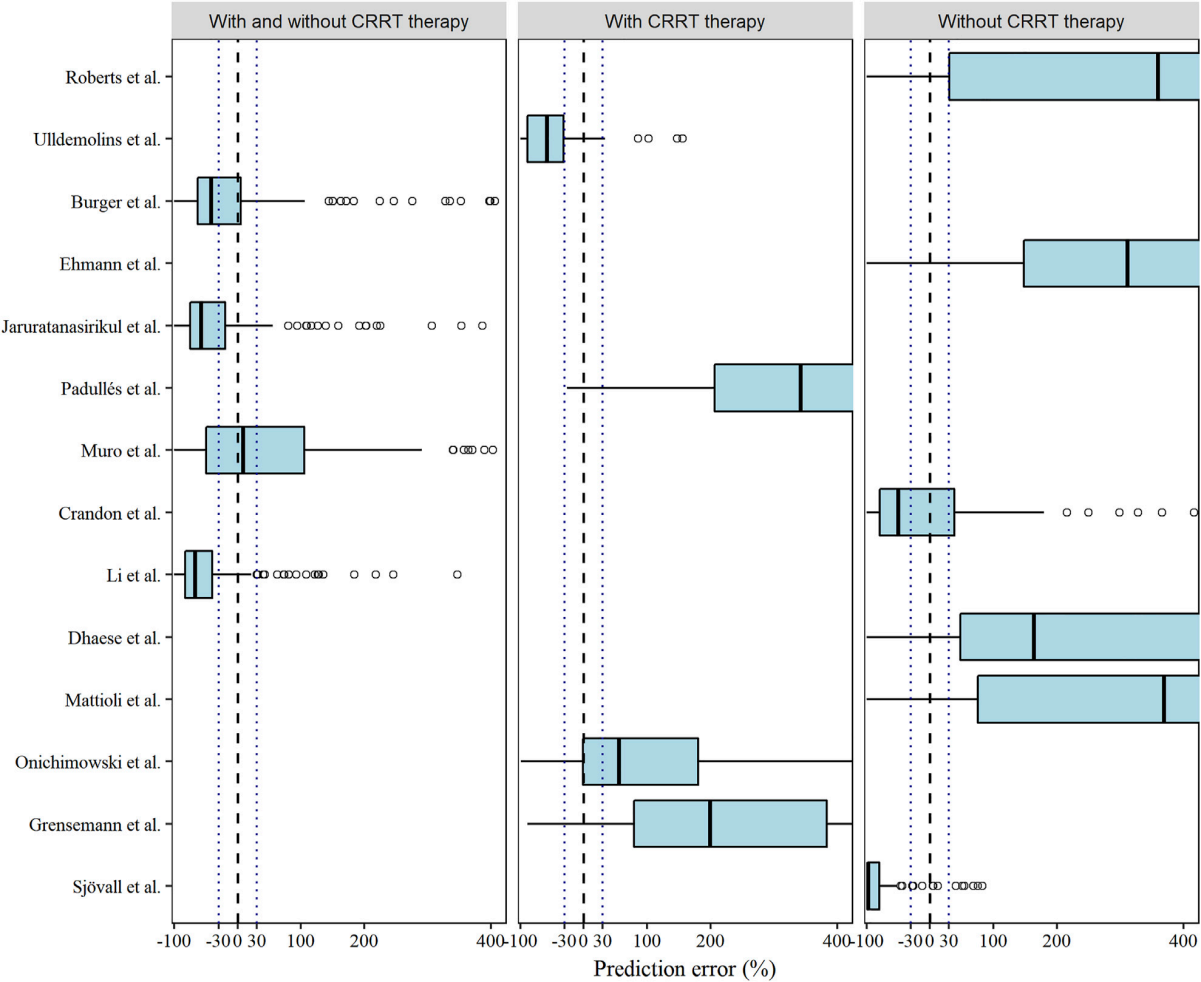
Box plots of the prediction error (PE %) of the studied meropenem models. Black dashed and dotted lines are reference lines indicating PE% of 0% or ±30%, respectively.

Among the models in the CRRT category and non-CRRT category, the Ulldemolins and Sjövall models displayed slight underestimations (Ulldemolins et al., 2015; Sjövall et al., 2018), and the Onichimowski model (Onichimowski et al., 2020) could be identified as a marginally better one, whereas rest (Roberts et al., 2009; Mattioli et al., 2016; Dhaese et al., 2019; Ehmann et al., 2019; Padullés Zamora et al., 2019; Grensemann et al., 2020; Onichimowski et al., 2020) showed obvious overpredictions of meropenem concentrations (Figure 2). Although none of the models showed compliance with the combined criteria of MDPE ≤ ±20%, MAPE ≤30%, F_20_ ≥ 35%, and F_30_ ≥ 50%, the models proposed by Muro et al. (2011), Crandon et al. (2011), and Burger et al. (2018) ranked as the top three models with slightly preferable values of F_20_ and F_30_. In particular, the Muro model showed the least bias, with an MDPE of 8.64% (Muro et al., 2011).

#### Simulation-Based Diagnostics

Similarly, with the exception of the model proposed by Crandon et al., the pcVPC plots of most CRRT and non-CRRT models (Roberts et al., 2009; Mattioli et al., 2016; Sjövall et al., 2018; Dhaese et al., 2019; Ehmann et al., 2019; Padullés Zamora et al., 2019; Grensemann et al., 2020; Onichimowski et al., 2020), displayed a remarkable trend in the overprediction of the typical population predictions and variability (Supplementary Figure S1). The models by Burger et al. (2018) and Jaruratanasirikul et al. (2015) showed relative superiority in the pcVPC plot. Although there was a slight inconsistency between the 95th percentiles of the observed data and the simulated intervals of the corresponding percentiles, represented by the highest dashed lines and the transparent blue areas, respectively, the 5th and 50th percentiles of the observed meropenem concentrations were mostly situated within the associated confidence intervals of the simulated data.

### Detailed Bayesian Forecasting Analysis (Individual Level)

#### Comparisons of Empirical Bayesian Estimation

**FIGURE 3:**
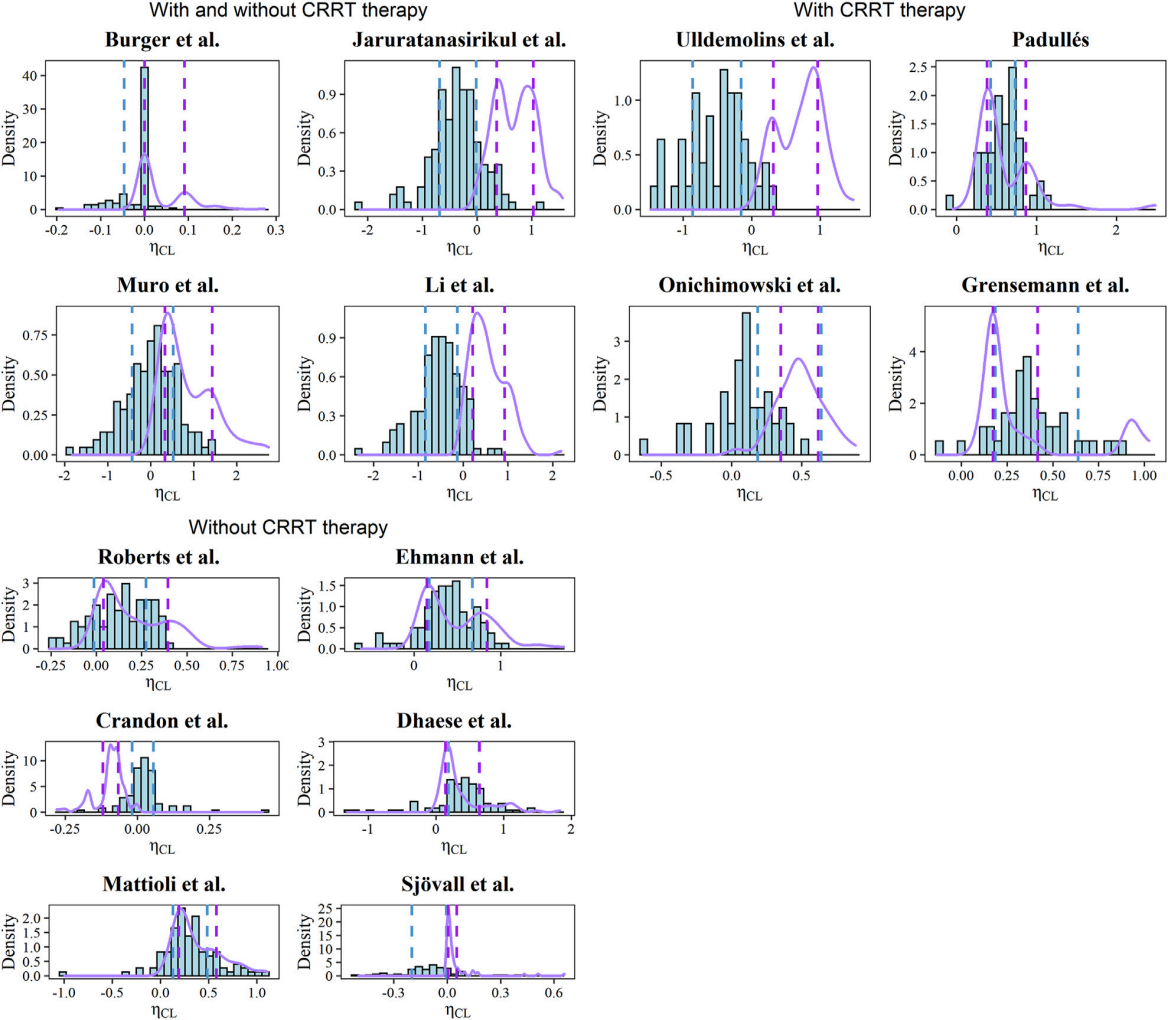
Histograms of EBEs for the CL of each model. The purple solid lines represent the density of simulated CL. The blue and purple dashed line represents the 20th and 80th percentiles of calculated and simulated EBEs, respectively.

The estimated and simulated distributions of CL after the post hoc analysis were plotted in Figure 3 with the corresponding typical values and interindividual variabilities. Due to the inaccessible IIV of the Vd in the Burger, Muro, and Dhaese models, the EBEs of Vd were not investigated (Muro et al., 2011; Burger et al., 2018; Dhaese et al., 2019). Diversified levels of variability of CL could be observed across the identified models, which can be explained by the various study designs and population differences. In the Mattioli model, the 20th and 80th percentiles of the simulated distribution were close to the corresponding percentiles of the estimated distribution (Mattioli et al., 2016).

#### Bayesian Forecasting

The predictive performance of the a priori method was highly variable among the models with the external dataset (n = 134), as revealed by the rBias ranging from −302.96 to 130.37% and the rRMSE ranging from 14.78 to 241.84% (Supplementary Table S2). In the graphical analysis, the Bayesian estimated method showed obvious superiority compared with the priori method, where most of the rBias were within the range of ±20% after the Bayesian approach (Supplementary Figure S2).

In a subset of the dataset (n = 13), the rBias and rRMSEs of the third meropenem concentrations were marginally decreased across the models using the combinations of previous observations (Supplementary Table S3). This was also reflected in the IPEs of the models after the Bayesian approach (Supplementary Figure S3). In addition, the model proposed by Crandon et al. (2011) almost lacked bias with two priors available (MDIPE = 15.6%, MAIPE = 35.8%, IF_20_ = 37.5%, and IF_30_ = 50%). Generally, one prior observation can significantly improve the predictive performance, and further priors would not necessarily imply further improvement. Therefore, the dosing occasion in subsequent dose tailoring steps was determined as the occasion with the most recent observation (Supplementary Figure S4).

**FIGURE 4:**
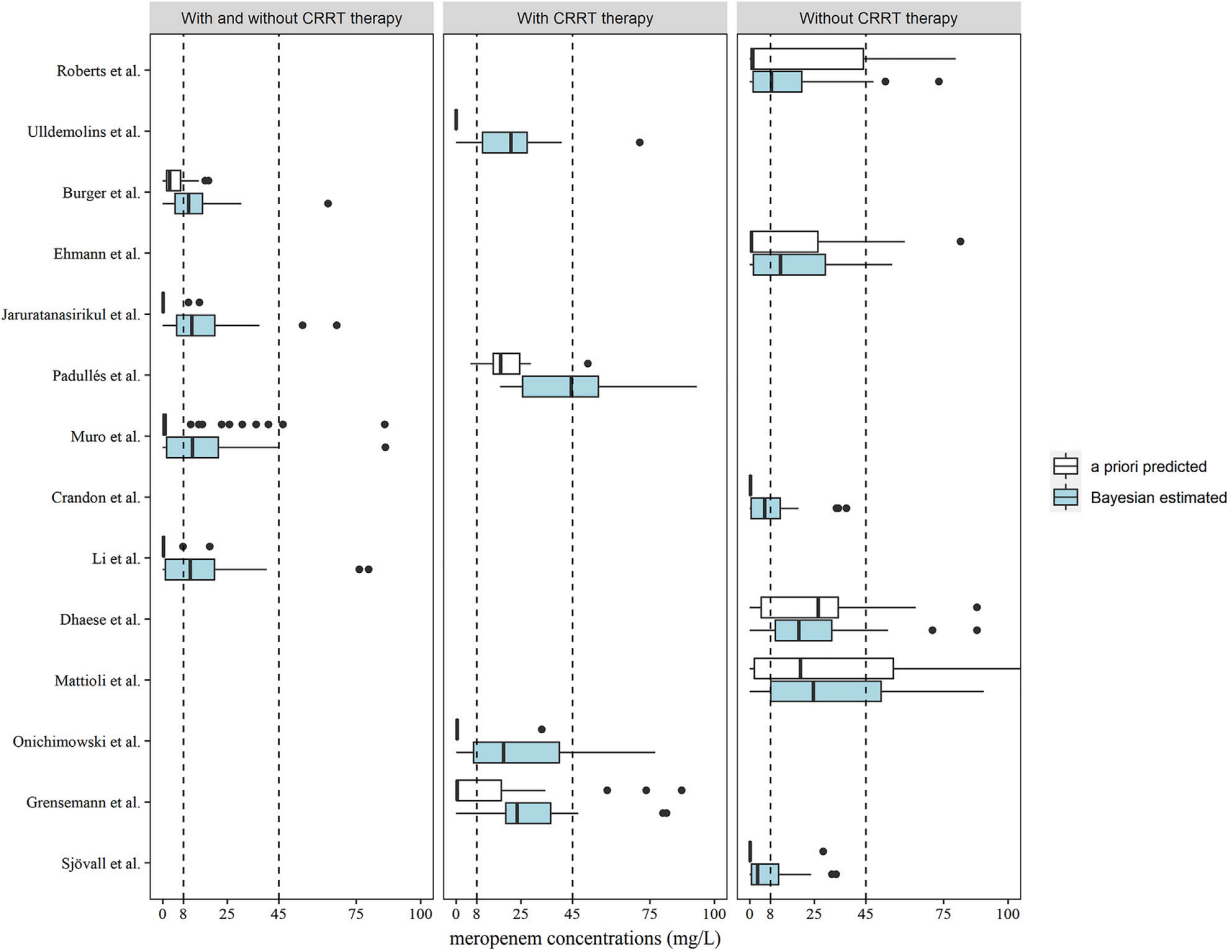
Box plots of predicted meropenem trough concentrations after a priori predicted (white) and Bayesian estimated (blue) methods (n = 46). Box plots represent the 25th, 50th, and 75th percentiles, and the outliers are marked as dots. The area enclosed by the black dotted line indicates the target meropenem concentration range of 8–45 mg/L.

The meropenem concentrations were predicted and estimated in patients with two or more observations available (n = 46) using a priori and Bayesian approaches (Figure 4). In the models developed by patients with and without CRRT therapy, more predicted concentrations were in the range of 8–45 mg/L compared to the a priori approach and the methods of Burger et al. (2018), Jaruratanasirikul et al. (2015), Muro et al. (2011), Li et al. (2006) in this category led to similar dose adjustments changes of 67.39, 58.70, 63.04, and 58.70%, respectively, of the observed concentrations (Table 3). In total, 12 out of 14 models showed the preferable capability of meropenem dose adjustment after the Bayesian approach, and the Ehmann model displayed a higher accuracy of 68.97% compared to other models for the non-CRRT category (Ehmann et al., 2019).

**TABLE 3:** Predictions of the need for dose adjustments based on the target of 100% ƒT>4×MIC (MIC = 2 mg/L) according to meropenem concentrations following the second occasion (n = 46) in a priori estimation and Bayesian estimation.

Models	A Priori Predicted								Bayesian Estimated					
**With and without CRRT therapy**														
**What dose adjustment is required?**	**Increase (n = 15)**		**Maintain (n = 25)**		**Decrease (n = 6)**			**Increase (n = 15)**		**Maintain (n = 25)**		**Decrease (n = 6)**		
**Correctly predicted?**	**Yes**	**No**	**Yes**	**No**	**Yes**	**No**	**Accuracy**	**Yes**	**No**	**Yes**	**No**	**Yes**	**No**	**Accuracy**
Burger et al.	13	2	7	18	0	6	43.48%	11	4	19	6	1	5	67.39%
Jaruratanasirikul et al.	15	0	1	24	0	6	34.78%	10	5	16	9	1	5	58.70%
Muro et al.	15	0	4	21	1	5	43.48%	11	4	16	9	2	4	63.04%
Li et al.	15	0	1	24	0	6	34.78%	11	4	16	9	0	6	58.70%

**With CRRT therapy**														
**What dose adjustment is required?**	**Increase** **(n = 1)**		**Maintain (n = 11)**		**Decrease (n = 3)**			**Increase (n = 1)**		**Maintain (n = 11)**		**Decrease (n = 3)**		
**Correctly predicted?**	**Yes**	**No**	**Yes**	**No**	**Yes**	**No**	**Accuracy**	**Yes**	**No**	**Yes**	**No**	**Yes**	**No**	**Accuracy**
Ulldemolins et al.	1	0	0	11	0	3	6.67%	0	1	8	3	1	2	60.00%
Padullés et al.	0	1	9	2	0	3	60.00%	0	1	7	4	2	1	60.00%
Onichimowski et al.	1	0	1	10	0	3	13.33%	0	1	5	6	1	2	40.00%
Grensemann et al.	0	1	0	11	0	3	0.00%	0	1	8	3	1	2	60.00%

**Without CRRT therapy**														
**What dose adjustment is required?**	**Increase** **(n = 11)**		**Maintain (n = 15)**		**Decrease (n = 3)**			**Increase (n = 11)**		**Maintain (n = 15)**		**Decrease (n =3)**		
**Correctly predicted?**	**Yes**	**No**	**Yes**	**No**	**Yes**	**No**	**Accuracy**	**Yes**	**No**	**Yes**	**No**	**Yes**	**No**	**Accuracy**
Roberts et al.	9	2	3	12	2	1	48.28%	8	3	9	6	2	1	65.52%
Ehmann et al.	7	4	4	11	2	1	44.83%	8	3	11	4	1	2	68.97%
Crandon et al.	11	0	0	15	0	3	37.93%	10	1	9	6	0	3	65.52%
Dhaese et al.	6	5	10	5	2	1	62.07%	4	7	11	4	2	1	58.62%
Mattioli et al.	3	8	2	13	2	1	24.14%	3	8	9	6	2	1	48.28%
Sjövall et al.	11	0	1	14	0	3	41.38%	10	1	7	8	0	3	58.62%

“What dose adjustment is required?” refers to the dose adaptations of increase, decrease, maintain based on the realistic meropenem observations, while “Correctly predicted?” refers to the accuracy the meropnenem dosing adaptations based on the predicted values when compared with the adjustments based on the realistic ones.

## Discussion

Although a number of external evaluation studies of meropenem have been published, it is still unclear whether these models can be extrapolated to Chinese patients, and how these models can be used appropriately for guiding individualized meropenem therapy. Wang et al. (2020) used external data from patients who received a continuous infusion of meropenem, which may be inapplicable to those models with intermittent infusion or bolus. Tamatsukuri et al. and Wong et al. assessed the applicability of the models in patients with distinct renal function classes, such as patients with augmented renal clearance (Wong et al., 2015; Tamatsukuri et al., 2018). Considering the potential correlation between renal function and meropenem pharmacokinetics, evaluation of the meropenem modes in a population ranging from CRRT/renal impairment to augmented renal clearance may be needed. This study sought to elucidate the heterogeneity of meropenem population pharmacokinetic models with respect to their capabilities of correctly predicting meropenem concentrations based on overall model evaluations and detailed Bayesian forecasting analysis. Owing to broader model testing and inclusion of patients covering the full spectrum of renal function, especially CRRT patients, in this analysis, the clinical utility was increased by covering the model used in these common kinds of critically ill patients. In addition, the comparisons of EBE between estimated and theoretical distributions (assessing model adequacy) and comparisons of dose adaptations between a priori approach and Bayesian methods (assessing model utility) were first applied to the external evaluation studies of meropenem, implying that further similar external validation studies should not only assess the predictability of the models, but also the model adequacy and clinical applicability.

Although none of the identified meropenem models passed all performance tests, the model proposed by Muro et al. (2011) showed the least bias in the PE% test, which was consistent with the previous study by Wong et al. (2015). The slightly superior performance of the model can be possibly explained by the racial and geographical dominance due to the Asian model-building population. This is because similar populations have less interethnic variability in pharmacological effects and metabolic polymorphisms (Fricke-Galindo et al., 2016). Furthermore, a modified serum creatinine concentration of 0.4 mg/L was set for creatinine concentrations lower than the cutoff value to avoid discrepancies between the serum creatinine concentrations and renal function, which may be another contributing factor to the global fit of the Muro model.

Our validation dataset represented diverse ICU patients of different ages (22–88 years old), Clcrs (3.4–271.5 ml/min), and underlying diseases. Since meropenem is predominantly excreted by the kidneys, the creatinine clearance or other predictors of renal function should imply an essential consideration of renal function when designing a meropenem dosage regimen. Ideally, Clcr values can be added for further subgroup analysis for more accurate results in this study. However, due to the lack of reported Clcr values in some identified models (Li et al., 2006; Ehmann et al., 2019; Grensemann et al., 2020) and the impact of CRRT on the pharmacokinetics of meropenem, whether the patients were treated with CRRT was used as a surrogate criterion. Though the dataset was divided into different subpopulations, inevitable heterogeneity was still a weakness of the analysis. For example, the study by Roberts et al. (2009) excluded patients with renal dysfunction and, therefore, may not be appropriate for describing the meropenem pharmacokinetics in patients with extensive renal function failure, as evidenced by the obvious overprediction of our results, and this implies that other important variants may be included for further subgroup analysis. Although the EBEs of the CL in the Mattioli model were close to the theoretical distribution, high prediction errors could be explained by the applied covariates (Mattioli et al., 2016). In fact, most of our sepsis patients did not fully overlap with the severity range of sepsis incorporated into the final model. In addition, significant variability of 44.38 and 66.48% in CL and Vd indicated further investigations of variation-causing factors. Besides, the different types of infusion could also lead to poor predictive performance, that is, continuous infusion applied in the model of Dhaese et al. differed from the continuous infusion and intermittent infusion in our dataset, which made it a biased one (Dhaese et al., 2019).

In this study, patient covariates were slightly helpful in predicting meropenem concentrations correctly, indicating challenges in the selection of initial meropenem dose using the model-based approach. Compared to the a priori predicted approach, the rBias and rRMSEs of the model predictions were marginally decreased based upon the Bayesian forecasting. In particular, better predictive performance could be achieved with the most recent meropenem observation with the Bayesian approach. Hence, the use of recent observations would be an efficient tool in subsequent dosing adaptations when combined with the Bayesian approach, as illustrated by the higher proportions of patients targeting the concentrations after the Bayesian analysis. Although the dose adjustments of the Ehmann model showed slightly higher accuracy of 68.97% of the time as determined by the meropenem observations, the poor predictive performance could limit its clinical applicability and generalizability to a wider population.

According to the stated results, four meropenem models developed in critically ill patients with CRRT therapy showed inadequate predictive performance (Ulldemolins et al., 2015; Padullés Zamora et al., 2019; Grensemann et al., 2020; Onichimowski et al., 2020). This is not surprising as no CRRT-related factors are retained in the final model. In fact, the pharmacokinetic and structural characteristics of meropenem make it a dialyzable drug owing to the main determinants of CRRT clearance (high affinity of water, high unbound fraction, and low volume of distribution). Thus, CRRT is supposed to be a multidimensional and continuous covariate to characterize the meropenem pharmacokinetics based on its diversified equipment options and hemodialysis methods. This also implies that factors relevant to the CRRT settings, such as the type of the membrane and dialysate flow, should be considered as much as possible when screening for the covariates in further population pharmacokinetic studies of CRRT patients.

Some limitations of the present study need to be considered. First, the Cockcroft–Gault formula used for Clcr estimation has reported variability in critically ill patients (Sunder et al., 2014). Since most of the published models estimated Clcr in the same way, little bias would occur. Second, considering single-center TDM data was used in the study, data quality and limited sample size may affect the assessments of model predictability. However, rigorous methods and stringent criteria applied in the present study may help to reduce the bias caused by the above limitations. Furthermore, since the ultimate objective of this analysis was to inform the precision dosing of meropenem in clinical settings with the published models, it was necessary to test them under more realistic conditions, such as the limited samples a patient can provide.

## Conclusion

To summarize, none of the identified popPK models could adequately describe meropenem pharmacokinetics in critically ill patients at our center, but use in Bayesian analysis may improve the prediction results. This study demonstrated that the selection of an initial meropenem dose with a priori prediction is challenging, but model-based dosing combined with the TDM process could be applied in clinical practice of meropenem therapy.

## Data Availability

The datasets presented in this article are not readily available because of ethical & legal restrictions. Requests to access the datasets should be directed to Qi Pei, peiqi1028@126.com.

## Abbreviation

ƒT > MIC, free plasma concentration above the minimum inhibitory concentration; CRRT, continuous renal replacement therapy; ICU, intensive care unit; popPK, population pharmacokinetics; TDM, therapeutic drug monitoring; PE, prediction error; PRED, predicted concentration; OBS, observation; MDPE, median prediction error; MAPE, median absolute prediction error; F_20_, the percentage of prediction error falling within 20%; F_30_, the percentage of prediction error falling within 30%; pcVPC, prediction-corrected visual predictive checks; NPDE, normalized prediction error; EBE, empirical Bayesian estimate; rBias, relative bias; rRMSE, relative root mean squared error; IPRED, individual predicted concentration; IPE, individual prediction error; MDIPE, median individual prediction error; MAIPE, median absolute individual prediction error; IF_20_, the percentage of individual prediction error falling within 20%; IF_30_, the percentage of individual prediction error falling within 30%; Clcr, creatinine clearance; PD, pharmacodynamic.
