# Supplementary material for: External Evaluation of Population Pharmacokinetic Models to Inform Precision Dosing of Meropenem in Critically Ill Patients
Source: Front Pharmacol. 2022 May 18;13:838205. doi: 10.3389/fphar.2022.838205 (PMC9157771; doi:10.3389/fphar.2022.838205)
Supplement: Supplementary file 1 [file DataSheet1.docx]

Supplementary Material

**1.1 Appendix S1*.*** Normalized prediction distribution error test of the published meropenem population pharmacokinetic models

The validity of the normalized prediction error test (NPDE) assumed values obeyed a normal distribution with a mean of zero and a variance of one. Therefore, a Wilcoxon signed-rank *t*-test for mean value, a Fisher test for variance, and a Shapiro–Wilk test for normality of the distribution were used to determine the NPDEs. A *p*-value < 0.05 was defined to assess the statistical significance. Additionally, a histogram of NPDE and scatter plots of NPDE versus predictions and time after dose were visually inspected to assess the mode fit.

The results below showed most NPDEs of the identified models did not follow a standard normal distribution, except for the Onichimowski model with a globally adjusted *p*-value of 0.32(Onichimowski et al., 2020). Although the distribution of the Muro model showed normality (Shapiro–Wilk test, *p* = 0.472), its variance was significantly different from the expected value of 1 (Fisher test, *p* = 1.18 × 10^-12^), indicating that simulations of meropenem had been inappropriately applied(Muro et al.)

**
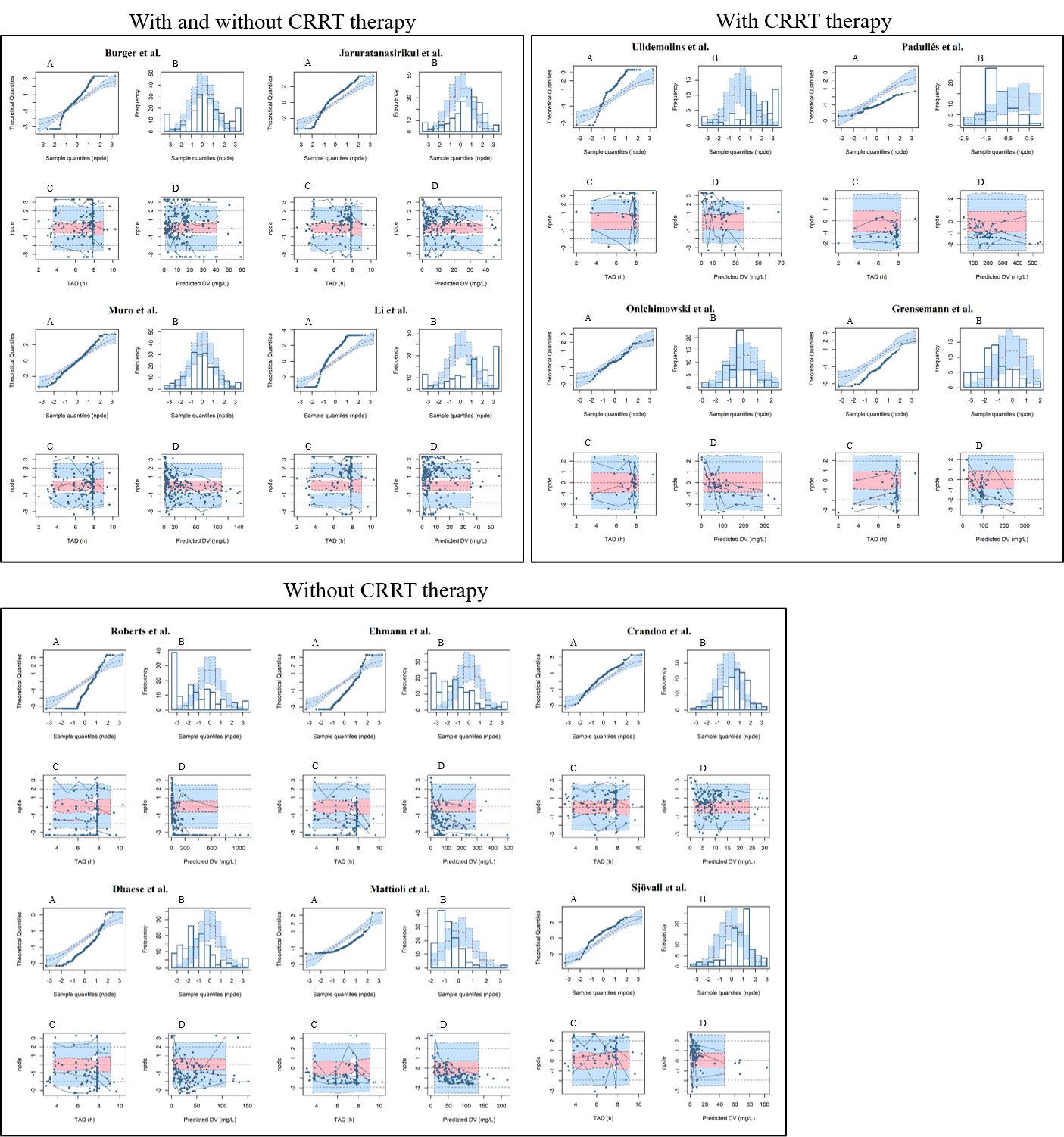
**

Panel A: Quantile - quantile plot of the distribution of the NPDE against the theoretical distribution (semitransparent blue fields), B: histogram of the distribution of the NPDE against the theoretical distribution (semitransparent blue fields), C: NPDE versus time after the last dose (h), and D: NPDE versus predicted concentrations (mg/L). In the last two plots, the blue points represent the NPDE of the observations. The solid line represents the 5th, 50th, and 95th percentiles of NPDE observations. The shaded areas represent the 95% confidence intervals around the simulated 5th, 50th, and 95th percentiles.

**1.2 Supplementary figures**

**
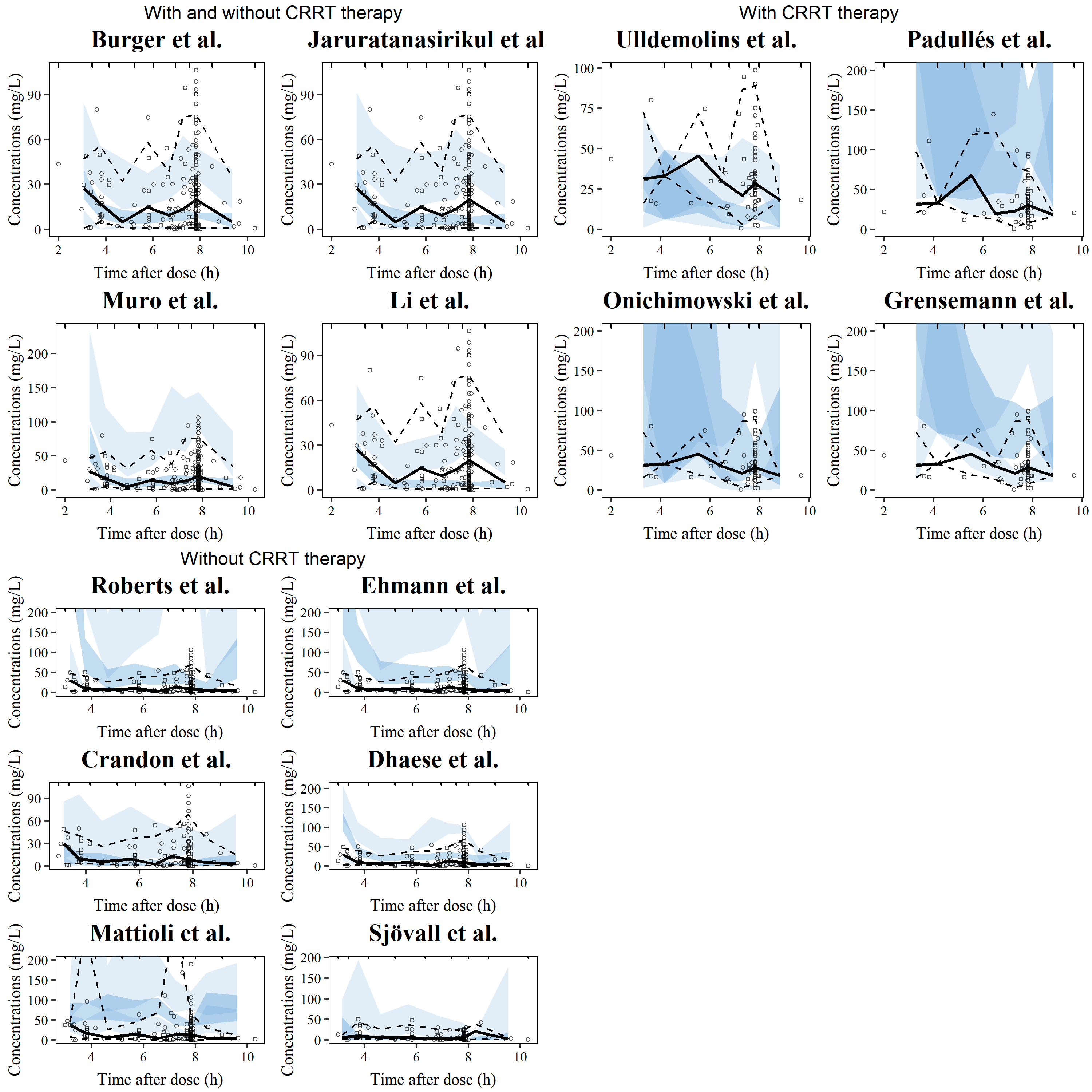
**

**Supplementary Figure S1.** Prediction-corrected visual predictive check plots for meropenem concentrations of the studied models. The measured concentrations of meropenem are marked as the black circles. The solid line and dotted lines represent the 5th, 50th, 95th percentiles of the meropenem concentrations in the external dataset, respectively, and semitransparent blue and blue fields represent simulation-based 90% confidence intervals for the 5th, 50th, 95th percentiles.

**
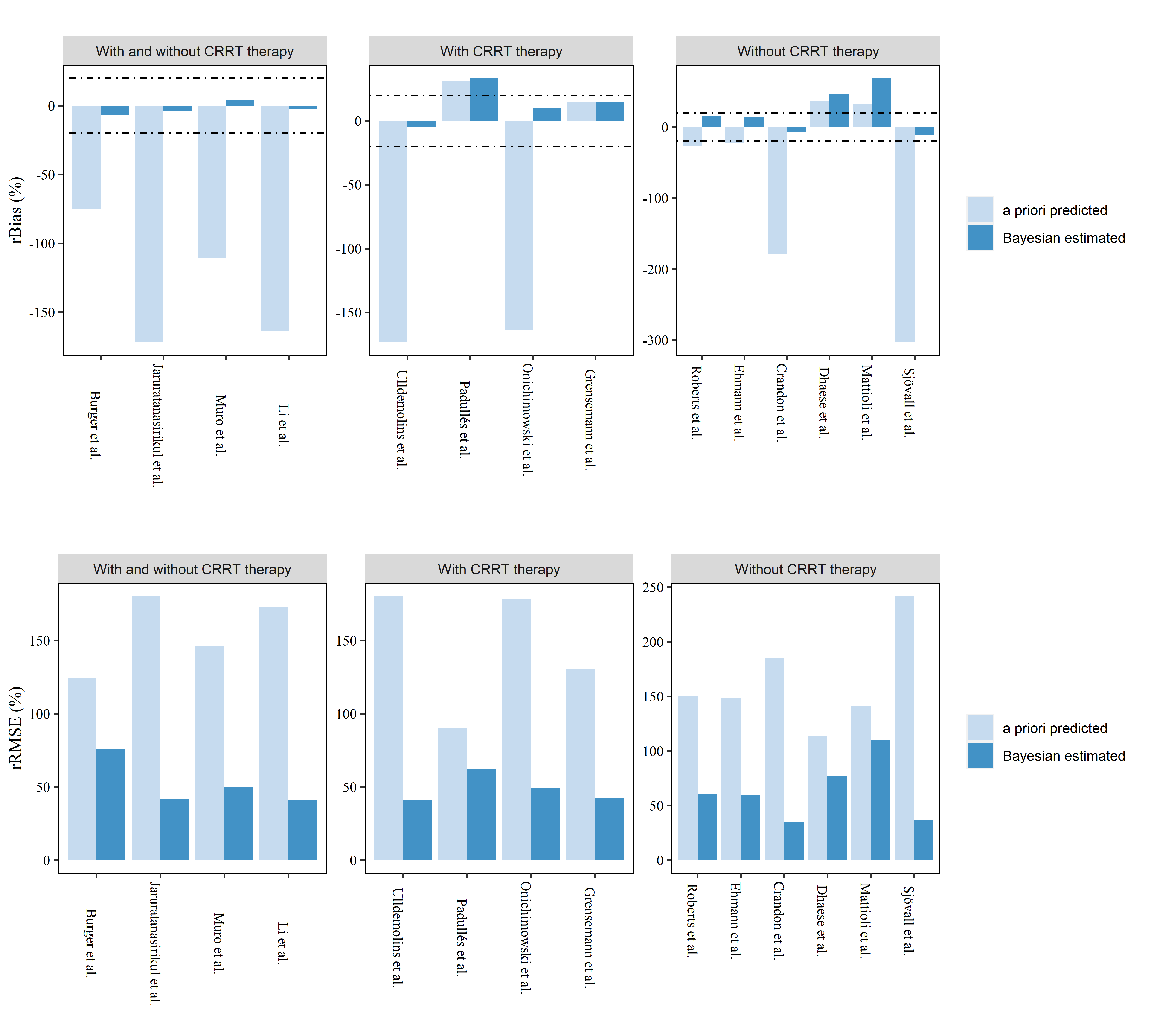
**

**Supplementary** **Figure S2.** The relative bias (rBias) and relative root mean squared error (rRMSE) of the predicted versus the observed meropenem concentrations after a priori predicted and Bayesian estimated method with the full dataset, respectively.

**
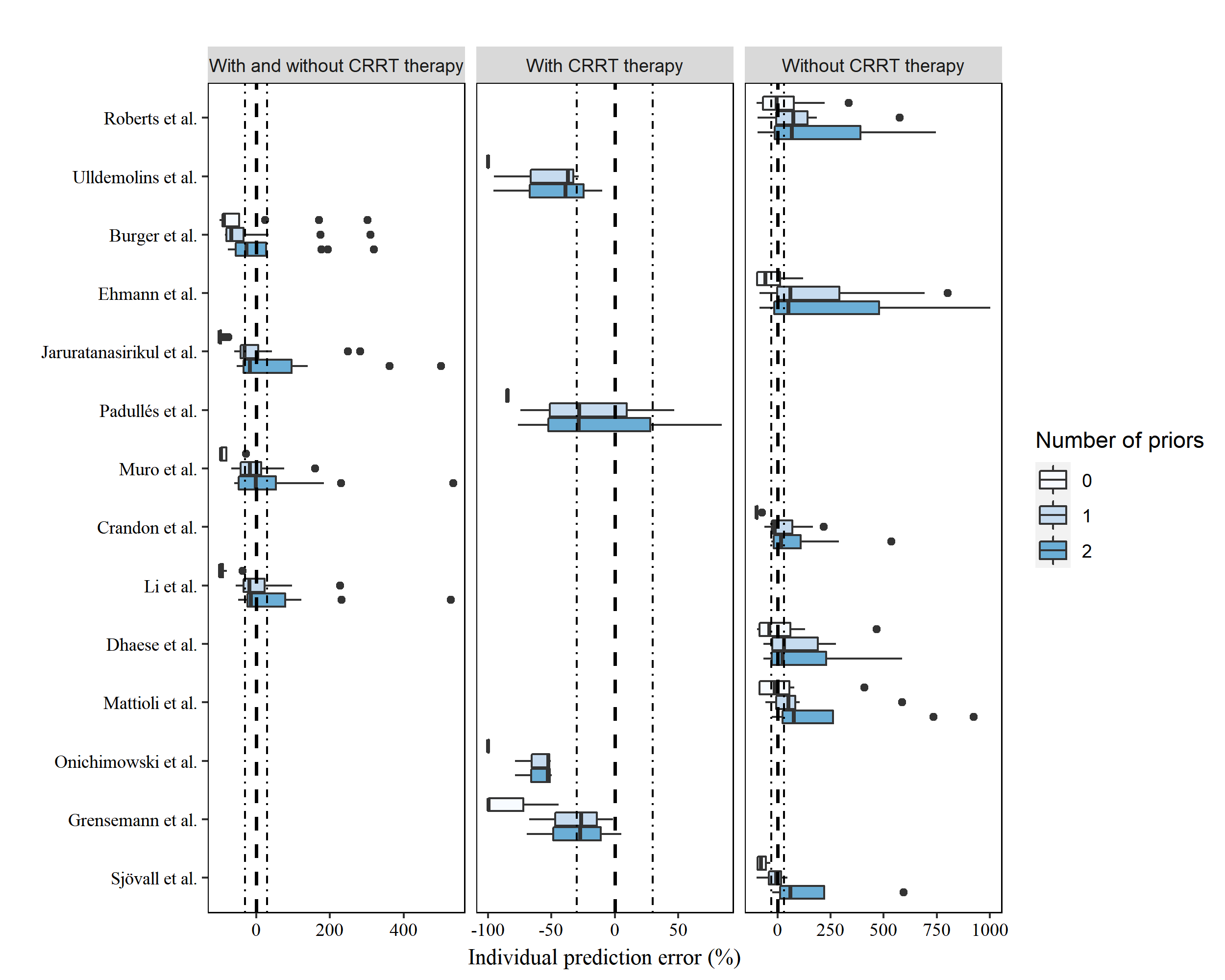
**

**Supplementary Figure S3.** Box plot of the individual prediction error (IPE%) after Bayesian forecasting following the third occasion in different scenarios (n = 13), whereas 0 represents predictions without priors, and 1 - 2 represent predictions with one or two prior information respectively. The black dashed and dotted lines are represented IPE of 0 and ± 30%, respectively.

**
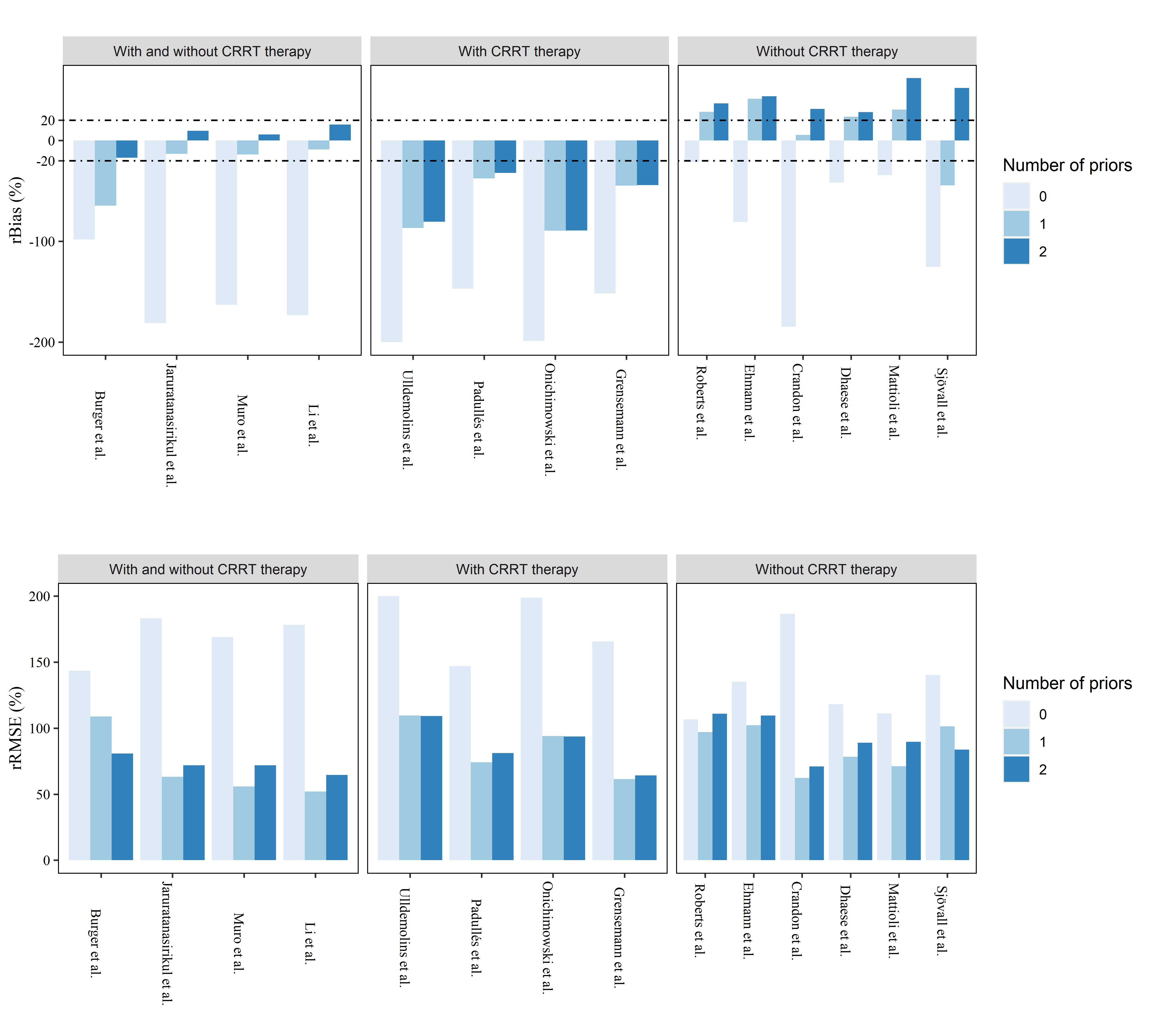
**

**Supplementary Figure S4.** The relative bias (rBias) and relative root mean squared error (rRMSE) of meropenem concentrations after Bayesian forecasting following the third occasion in different scenarios (n = 13), whereas 0 represents predictions without priors, and 1 - 2 represent predictions with one or two prior information respectively. rBias of ± 20% is plotted as the black dotted lines.

**1.3 Supplementary tables**

**Supplementary Table S1.** Demographic characteristics of published population pharamcokinetic models of meropenem.

| **Study**  **(publication year)** | **Country**  **(single/**  **multiple sites)** | **Study design** | **Subject**  **(age)** | **Number of patients (male /**  **female)** | **Sampling schedule (Number of samples)** | **Dosing** | **Weight**  **(kg)** | **Crcl**  **(mL/min)** | **Assay method** |
| --- | --- | --- | --- | --- | --- | --- | --- | --- | --- |
| Roberts et al. (2009)^c^ | Australia (single) | NA | adult critically ill patient with sepsis and normal renal function   1. bolus infusion:   55(48–61)   1. continuous infusion: 57(54–63) | 10 (7/3) | Day1: 0, 3, 5, 7, 10 15, 20, 30, 45, 60, 90, 150, 240, 360, 480 min  Days 2-5: 0, 5, 30, 60, 120, 180, 240, 360, 480 min  (222 plasma and 274 microdialysis) | bolus infusion:  1.5g+1gQ8hcontinuous infusion:  0.5g+3gQd | bolus infusion:  80(75-85)  continuous infusion: 75(75–85) | bolus infusion: 106 (98–127)  continuous infusion:  93 (69–161) | plasma:  HPLC-UV;  microdialysis: HPLC-MS/MS |
| Ulldemolins et al. (2015)^d^ | Spain (multiple) | prospective,  open-label | critically ill adult patients with septic shock and CRRT  66.5(34-85) | 30 (16/14) | (1) at 10min-predose, 0, 15, 60min, between 3-6h and just before the next dose.  (2) at 10min-predose, 0, 60, 120min, and before the next dose  (153) | 0.5-2g  Q6h-Q12h | 72.8  (49- 126) | NA | HPLC-MS/MS |
| Burger et al.(2018) | Switzerland(single) | prospective | critically ill adult patients  63 (49–70) | 101 (57/44) | C_0_, C_1_, C_8-12_, others  (380 blood sample and 129 filtrate–dialysate samples) | NA | 72  (58–85) | <130: n=11  90-129: n=7  60-90: n=14  30-60: n=17  15-30: n=7  <15: n=1 | UPLC-MS,  HPLC-UV |
| Ehmann et al.(2019)^c^ | Germany  (single) | prospective,  observational | critically ill patients with severe infections and non-CRRT | 48 (NA) | NA(1376) | 1, 2g Q8h | NA | NA | NA |
| Jaruratanasirikul et al. (2015) | Thailand  (single) | NA | critically ill adult patients with severe sepsis or septic shock  57.22 (33-83) | 9 (8/1) | 0, 0.25, 0.5, 1, 1.25, 1.5, 2, 2.5, 3, 4, 5, 8, 8.5, 9, 9.5, 10, 12, 14, 16 h  (171) | 1g Q8h | 62.88  (49-80.5) | 78.42  (12.37-214.55)^a^ | HPLC-DAD |
| Padullés et al.(2019)^d^ | Spain  (single) | prospective,  open-label, observational | sepsis adult patients undergoing CRRT  70 (34-80) | 12 (8/4) | 0, 0.5, 0.75, 1, 2, 4, 8, 16, 24h  (108) | 1g Q8h | 79 (60-110) | NA | HPLC-MS/MS |
| Muro et al.(2011) | Japan  (single) | NA | adult patients  71.5(25-91) | 68 (NA) | after the third dose interval  (68) | NA | 52.1  (30.7-107) | 65.5 (8.8-406) | HPLC-UV |
| Crandon et al.(2010)^c^ | America  (single) | NA | medical, cardiothoracic, surgical, and neurotrauma ICU patients  60.0 ± 17.5 | 21 (12/9) | after at least three  consecutive doses  (55) | 0.5-2g Q8h,  0.5g Q6h | 88.9 ± 22.3 | 70 (35–201) | HPLC-UV |
| Li et al. (2006) | America  (multiple) | NA | intra-abdominal infections, community-acquired pneumonia, or ventilator-associated pneumonia patients  35 (18-93) | 79 (61/18) | at the third dose interval or steady state  (341) | 0.5, 1, 2g Q8h | 70  (40.6-127) | NA | HPLC-UV |
| Dhaese et al.(2019)^c^ | Belgium  (single) | prospective | surgical ICU adult patients  54 ± 15.4 | 21 (13/8) | after a minimum of 6h of meropenem therapy  (38) | Clcr <15:  1g + 1gQd;  Clcr15-29:  1g + 2gQd;  Clcr>30:  1g + 3gQd | 88.3 ± 17.6 | 74.9 ± 61.8 | UPLC-MS/MS |
| Mattioli et al.(2016)^c^ | America  (single) | prospective | patients with sepsis, severe sepsis, or septic shock  62 ± 12 | 27 (17/10) | immediately after the end of infusion; 1, 3, and 5 h; immediately before the next administration  (118) | 1,2g Q8,12 h | 76.2 ± 30.3 | 87.4 ± 44.2 | HPLC |
| Onichimowski et al. (2020)^d^ | Poland  (single) | prospective, observational cohort study | critically ill adult patients undergoing CRRT  67 (36-79) | 19 (14/5) | 0, 15, 30, 45, 60, 75, 90, 120, 180, 240 and 480 min  (256) | 1g Q8h | 80 (60-100) | 1.55 (0.6-3.7) mg/dL^b^ | HPLC |
| Grensemann et al. (2020)^d^ | Germany | open-label  prospective,  observational | patients with ACLF and  patients without ACLF (NLF)  ACLF: 59 (46-68)  NLF: 61 (55-75) | 19 (14/5) | 0, 1, 2, 4, 6 and 8 h after the start of infusion, 24, 25, 48 49h  (180) | 1g Q8h | ACLF:  78 (60-81)  NLF:  77 (55-86) | NA | HPLC-DAD |
| Sjövall et al.(2018)^c^ | Denmark  (single) | prospective, observational | patients with septic shock  64 (24-89) | 50 (26/24) | pre-dose, 30, 45min, 1, 2, 4 and 8 h  (350) | 1, 2g Q8h  1g Q12h | NA | 67 (7-204) | HPLC-MS/MS |

HPLC-UV, high-performance liquid chromatography with a UV detector; HPLC-MS/MS, high-performance liquid chromatography with tandem mass spectrometry; CRRT, continuous renal replacement therapy; NA, not available; UPLC-MS, Ultra-performance liquid chromatography with tandem mass spectrometry; DAD, Diode array detector.

^a^Calculated from serum creatinine using the Modification of Diet in Renal Disease equation.

^b^serum creatintine.

^c^Non-CRRT models.

^d^CRRT models.

**Supplementary Table S2.** The relative bias (rBias) and relative root mean squared errors (rRMSE) of meropenem observations using a priori prediction and Bayesian approach across the studied models.

| **Models** | **rBias (%)** | | **rRMSE (%)** | |
| --- | --- | --- | --- | --- |
|  | a priori predicted | Bayesian estimated | a priori predicted | Bayesian estimated |
| **With and without CRRT therapy** | | | | |
| Burger et al. | -75.15 | -6.90 | 124.32 | 75.71 |
| Jaruratanasirikul et al. | -171.80 | -3.80 | 180.26 | 42.00 |
| Muro et al. | -110.89 | 3.91 | 146.55 | 49.68 |
| Li et al. | -163.54 | -2.48 | 172.87 | 41.14 |
| **With CRRT therapy** | | | | |
| Ulldemolins et al. | -173.35 | -4.94 | 180.40 | 41.25 |
| Padullés et al. | 31.25 | 33.51 | 90.17 | 62.24 |
| Onichimowski et al. | -163.80 | 10.12 | 178.39 | 49.60 |
| Grensemann et al. | 130.37 | 14.94 | 14.78 | 42.39 |
| **Without CRRT therapy** | | | | |
| Roberts et al. | -26.13 | 15.05 | 150.60 | 60.77 |
| Ehmann et al. | -23.23 | 14.63 | 148.55 | 59.58 |
| Crandon et al. | -179.34 | -7.03 | 184.94 | 34.99 |
| Dhaese et al. | 36.46 | 46.83 | 113.84 | 76.97 |
| Mattioli et al. | 32.05 | 68.89 | 141.32 | 110.11 |
| Sjövall et al. | -302.96 | -11.63 | 241.81 | 36.75 |

**Supplementary Table S3**. The relative bias (rBias) and relative root mean squared error (rRMSE) of the predicted versus the observed meropenem concentrations after Bayesian forecasting following the third occasion in different scenarios, whereas 0 represents predictions without priors, and 1 - 2 represent predictions with one or two prior information respectively.

| **Models** | **rBias (%)** | | | **rRMSE (%)** | | |
| --- | --- | --- | --- | --- | --- | --- |
|  | P_0_ | P_1_ | P_2_ | P_0_ | P_1_ | P_2_ |
| **With and without CRRT therapy** | | | | | | |
| Burger et al. | -98.21 | -64.52 | -17.19 | 143.54 | 108.78 | 80.77 |
| Jaruratanasirikul et al. | -181.03 | -13.17 | 9.59 | 183.12 | 63.15 | 71.91 |
| Muro et al. | -162.88 | -13.80 | 6.05 | 168.91 | 55.87 | 71.86 |
| Li et al. | -173.24 | -8.92 | 15.72 | 178.16 | 51.93 | 64.57 |
| **With CRRT therapy** | | | | | | |
| Ulldemolins et al. | -200.00 | -86.71 | -80.47 | 200.00 | 109.56 | 109.16 |
| Padullés et al. | -146.95 | -37.59 | -32.21 | 147.00 | 74.11 | 81.07 |
| Onichimowski et al. | -198.74 | -89.54 | -89.26 | 198.74 | 94.00 | 93.74 |
| Grensemann et al. | -151.57 | -44.70 | -44.36 | 165.76 | 61.47 | 64.23 |
| **Without CRRT therapy** | | | | | | |
| Roberts et al. | -21.49 | 28.27 | 36.80 | 106.57 | 96.94 | 111.02 |
| Ehmann et al. | -80.80 | 41.35 | 43.79 | 135.22 | 102.26 | 109.59 |
| Crandon et al. | -184.71 | 5.43 | 31.30 | 186.57 | 62.26 | 70.93 |
| Dhaese et al. | -41.68 | 23.63 | 28.06 | 118.11 | 78.36 | 88.93 |
| Mattioli et al. | -34.51 | 30.56 | 61.82 | 111.17 | 71.12 | 89.68 |
| Sjövall et al. | -125.19 | -44.59 | 51.96 | 140.17 | 101.29 | 83.70 |
